# A Direct ALAD–SSUII Interaction Implies a Potential Link Between Tetrapyrrole and Terpenoid Pathways Toward Chlorophyll Biosynthesis in Plants

**DOI:** 10.3390/ijms27104225

**Published:** 2026-05-09

**Authors:** Na Huang, Zihan Wang, Shuyan Song, Yufan Chen, Peiwen Nian, Fei Zhou, Shan Lu

**Affiliations:** School of Life Sciences, Nanjing University, Nanjing 210023, China

**Keywords:** δ-aminolevulinic acid dehydratase (ALAD), geranylgeranyl diphosphate synthase (GGPPS), protein–protein interaction, GGPPS small subunit, tetrapyrrole pathway, chlorophyll biosynthesis

## Abstract

Chlorophylls are the major light-harvesting pigments in photosynthetic organisms. Their biosynthesis requires the coordinated supply of metabolic intermediates from two independent upstream branches: the methylerythritol 4-phosphate (MEP)-derived terpenoid pathway, which supplies the phytyl side chain via geranylgeranyl diphosphate (GGPP), and the tetrapyrrole biosynthesis pathway (TBP), which provides the porphyrin ring. How flux through these two branches is coordinated remains poorly understood. In this study, we report the identification of a direct protein–protein interaction between δ-aminolevulinic acid dehydratase (ALAD), the second enzyme of the TBP, positioned immediately upstream of the first metabolic branch point, and the Type II small subunit of GGPP synthase (SSUII), a key regulator of terpenoid flux toward chlorophyll biosynthesis. ALAD was identified as a candidate SSUII-interacting protein by co-immunoprecipitation coupled with LC-MS analysis of rice leaf tissue, with a sequence coverage of 57.04%. The interactions between OsALAD1 and OsSSUII in rice, and between AtALAD1 and AtSSUII in *Arabidopsis thaliana*, were validated by yeast two-hybrid assay and bimolecular fluorescence complementation (BiFC) in Arabidopsis protoplasts. BiFC imaging demonstrated that the interaction is localized to the chloroplast. Sequence analysis revealed that plant ALAD proteins are highly conserved, with 92% similarity between OsALAD1 and AtALAD1, and 76.9% similarity between OsALAD1 and the green alga *Chlamydomonas reinhardtii* CrALAD1, indicating cross-species conservation of the ALAD–SSUII interaction. In vitro enzyme activity assays showed that AtSSUII does not directly alter AtALAD1 catalytic activity, suggesting the interaction operates through post-translational rather than direct catalytic mechanisms. Overexpression of *AtALAD1* caused severe chlorosis and seedling lethality, while *AtSSUII* overexpression produced no distinct phenotype; neither transgene altered the transcript level of the other. Together, our results reveal a conserved cross-pathway protein–protein interaction linking the terpenoid regulatory machinery to the early TBP, suggesting a molecular possibility for the coordinated regulation of chlorophyll biosynthesis.

## 1. Introduction

**Figure 1:**
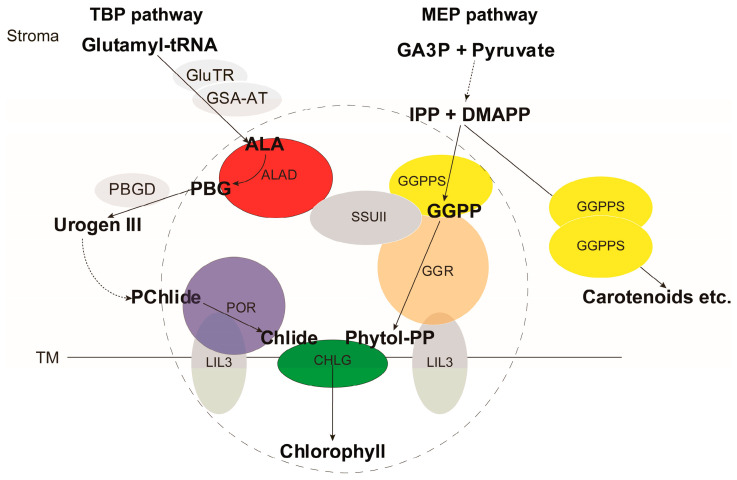
Scheme of the chlorophyll biosynthetic pathway. Enzymes are shaded, and metabolites are in bold-face. The dashed line indicates multiple metabolic steps. Proteins in the dash-lined circle are proposed to form protein complex through protein–protein interactions. Abbreviations are as follows: ALA, δ-aminolevulinic acid; ALAD, ALA dehydratase; CHLG, chlorophyll synthase; Chlide, chlorophyllide; DMAPP, dimethylallyl diphosphate; GA3P, glyceraldehyde 3-phosphate; GGPP, geranylgeranyl diphosphate; GGPPS, GGPP synthase; GGR, geranylgeranyl reductase; GluTR, glutamyl-tRNA reductase; GSA-AT, glutamate 1-semialdehyde aminotransferase; IPP, isopentenyl diphosphate; LIL3, light-harvesting like protein 3; MEP, methylerythritol 4-phosphate; PBG, porphobilinogen; PBGD, PBG deaminase; PChlide, protochlorophyllide; Phytol-PP, phytyl diphosphate; POR, protochlorophyllide oxidoreductase; TM, thylakoid membrane; SSUII, GGPPS small subunit protein; Urogen III, uroporphyrinogen III.

Chlorophyll biosynthesis in plants requires the concerted supply of metabolic intermediates from two distinct upstream pathways: the tetrapyrrole biosynthesis pathway (TBP) and the methylerythritol 4-phosphate (MEP)-derived terpenoid pathway [1]. Recent advances have further revealed the intricate regulatory networks governing chlorophyll biosynthesis, including transcriptional, post-translational, and metabolite-mediated mechanisms that fine-tune pigment accumulation in response to developmental and environmental signals [2,3]. Most of the enzymes in the chlorophyll biosynthetic pathway and their encoding genes have been elucidated from various plants and even bacteria [4,5]. In general, the biosynthesis of the tetrapyrrole ring starts from glutamyl-tRNA, which is converted to δ-aminolevulinic acid (ALA) by glutamyl tRNA reductase (GluTR) and glutamate-1-semialdehyde aminotransferase (GSA-AT) as the rate-limiting step of the entire pathway [6]. ALA dehydratase (ALAD) is a homo-octameric metalloenzyme that catalyzes the condensation of two ALA molecules to form porphobilinogen (PBG) [7]. ALAD activity in plants has been shown to be subject to redox regulation via thiol-based mechanisms, and its tight control is critical to prevent the accumulation of photoreactive tetrapyrrole intermediates that can generate singlet oxygen under illumination [8,9]. Four molecules of PBG are further processed by PBG deaminase (PBGD) to form linear tetrapyrrole, which is then cyclized into uroporphyrinogen III (Urogen III). A series of enzymes catalyze the conversion from Urogen III to protochlorophyllide (PChlide), which is reduced by NADPH:PChlide oxidoreductase (POR) to Chlide [4,10]. In parallel, the MEP pathway produces simultaneously isopentenyl diphosphate (IPP) and dimethylallyl diphosphate (DMAPP), which are condensed into C20 geranylgeranyl diphosphate (GGPP) by GGPP synthase (GGPPS). Geranylgeranyl reductase (GGR) reduces GGPP to phytyl diphosphate (Phytol-PP), and chlorophyll synthase (CHLG) esterifies the phytol chain onto Chlide to produce chlorophyll (Figure 1) [4,11].

Recently, we demonstrated in rice that a GGPPS small subunit protein (OsSSUII/OsGRP) shares high sequence similarity with GGPPS (OsGGPPS) but is catalytically inactive [12]. Through protein–protein interaction, OsSSUII recruits OsGGPPS from the stroma to the thylakoid membrane, where a protein complex also containing POR, CHLG, and the anchor protein LIL3 was identified [12,13]. This interaction demonstrates how the two upstream branches merge at CHLG in thylakoid membranes, anchored by the LIL3-GGR interaction [13]. Because GGPP is also a substrate for synthesizing other crucial metabolites such as carotenoids and gibberellins, such a mechanism secures the supply of GGPP for chlorophyll biosynthesis [12,14].

Despite extensive research on individual biosynthetic enzymes, the dynamic regulation connecting the two upstream metabolic branches for chlorophyll biosynthesis remains poorly understood. Here, we report the identification of a conserved protein–protein interaction between ALAD and SSUII, suggesting a previously unrecognized mechanism for coordinating flux through these two essential biosynthetic pathways. This finding opens new perspectives on chlorophyll biosynthesis regulation and provides insights into potential strategies for metabolic engineering to enhance photosynthetic performance in plants.

## 2. Results

### 2.1. OsALAD1 Is Identified as an OsSSUII-Interacting Protein by Co-IP/LC-MS

**Figure 2:**
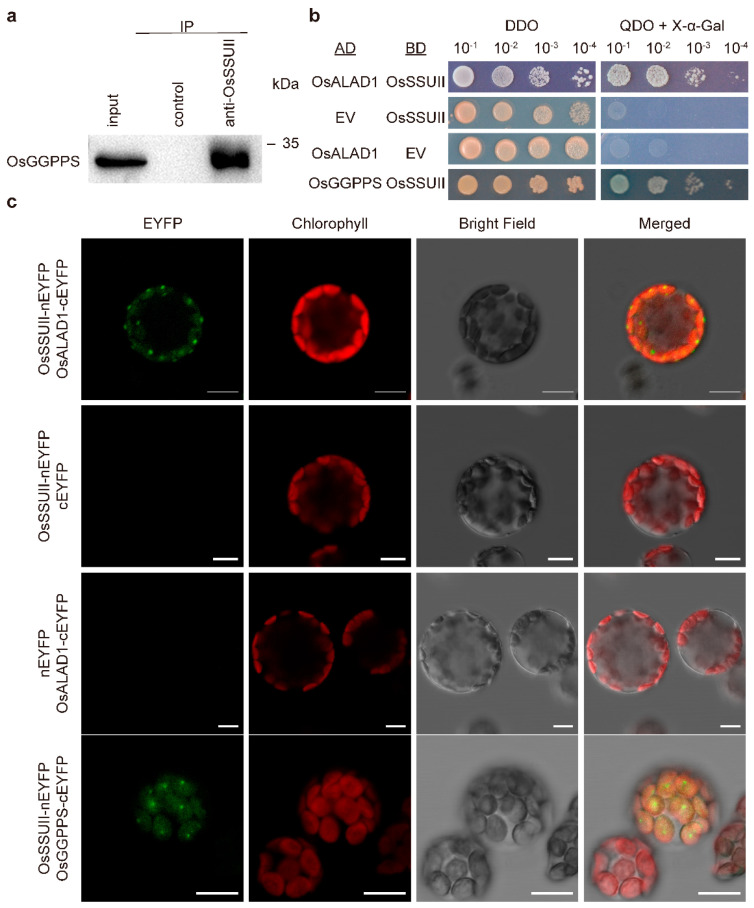
OsALAD1 interacts with OsSSUII. (**a**) Immunoblot detection of OsGGPPS from proteins co-precipitated with OsSSUII to validate the Co-IP strategy. Total proteins extracted from rice leaves were precipitated with the antiserum against OsSSUII and Protein A/G beads. The pelleted proteins were resolved by SDS-PAGE, followed by immunodetection using an antibody against OsGGPPS. Pre-immune serum was used as a negative control. (**b**) Yeast two-hybrid detection. Yeast cells harboring both constructs were spotted on non-selective (DDO) and selective (QDO) medium supplied with X-α-Gal. AD, activation domain of pGAD-T7; BD, DNA-binding domain of pGBK-T7; EV, empty vector. (**c**) BiFC detection in Arabidopsis leaf protoplasts. Co-expressed fusion constructs are indicated. Reconstituted EYFP fluorescence co-localizes with chlorophyll autofluorescence, indicating the interaction occurs in the chloroplast. Scale bars, 10 μm. For (**b**,**c**), known interaction between rice OsGGPPS and OsSSUII was used as positive controls.

After we previously established the protein–protein interaction between OsSSUII and OsGGPPS [12], we sought to determine whether OsSSUII also interacts with other proteins. We performed a co-immunoprecipitation (Co-IP) assay coupled with liquid chromatography–mass spectrometry (LC-MS) analysis. An antiserum raised against OsSSUII was used to precipitate proteins from total protein extracts prepared from 4-week-old rice leaves, using Protein A/G agarose beads [12]. Pre-immune serum served as the negative control. To validate the Co-IP strategy, co-precipitated proteins were first resolved by SDS-PAGE and subjected to immunoblotting with an antibody raised against OsGGPPS; a distinct immunoreactive band was detected in the anti-OsSSUII precipitate but not in the pre-immune serum control (Figure 2a), confirming the specificity of the Co-IP. Co-precipitated proteins were then subjected to LC-MS analysis.

Peptide fragments representing at least 18 proteins were detected from the Co-IP sample (Table 1). Importantly, the rice δ-aminolevulinic acid dehydratase (OsALAD1; UniProt ID Q5Z8V9; sequence coverage 57.04%) was identified among the co-precipitated proteins (Table 1). ALAD catalyzes the second step of the TBP, immediately downstream of the rate-limiting GluTR step, and its position upstream of the first metabolic branch point of the TBP makes it a potentially significant regulatory target for cross-pathway coordination. This raised our interest to analyze ALAD as a candidate SSUII-interacting protein.

### 2.2. Validation of the OsSSUII–OsALAD1 Interaction by Y2H and BiFC

To verify the interaction between OsSSUII and OsALAD1 identified by Co-IP/LC-MS, we first performed a pairwise yeast two-hybrid (Y2H) assay. Truncated peptides of OsALAD1 and OsSSUII with their predicted chloroplast transit peptides (cTPs) removed were fused to the activation domain (AD) and DNA-binding domain (BD), respectively. Yeast (*Saccharomyces cerevisiae*) AH109 cells co-expressing OsALAD1-AD and OsSSUII-BD grew robustly on stringent selective quadruple dropout (QDO; SD/–Leu/–Trp/–His/–Ade) medium supplemented with X-α-Gal and produced blue colonies, demonstrating a positive protein–protein interaction between OsALAD1 and OsSSUII (Figure 2b). All negative control combinations failed to grow on QDO medium. The previously validated OsGGPPS–OsSSUII interaction served as a positive control [12].

We subsequently performed a BiFC assay to confirm the interaction in a plant cellular context and to determine its subcellular localization. OsSSUII and OsALAD1 were fused to the N- and C-terminal halves of enhanced yellow fluorescent protein (nEYFP and cEYFP), respectively, and co-expressed in protoplasts prepared from *Arabidopsis thaliana* mesophyll cells. Reconstituted EYFP fluorescence was clearly detected when both fusion proteins were co-expressed, and the signal co-localized with chlorophyll autofluorescence, confirming that the OsSSUII–OsALAD1 interaction occurs within the chloroplast (Figure 2c). No fluorescent signal was detectable from any of the negative control combinations expressing one fusion protein or empty vector controls (Figure 2c).

### 2.3. The ALAD–SSUII Interaction Is Conserved Across Plant Species

**Figure 3:**
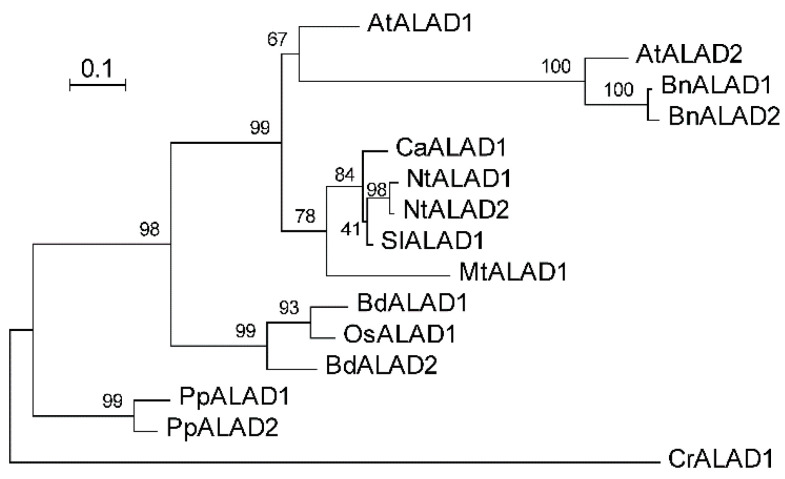
Phylogenetic analysis of plant ALADs. Sequences of ALAD ortholog proteins from different species were identified from UniProt (Table S1). A bootstrap (1000 replicates) Maximum-Likelihood phylogenetic tree was generated using IQ-TREE with the JTT+G4 model. Abbreviations before enzyme names represent their species (At, *Arabidopsis thaliana*; Bd, *Brachypodium distachyon*; Bn, *Brassica napus*; Ca, *Capsicum annuum*; Cr, *Chlamydomonas reinhardtii*; Mt, *Medicago truncatula*; Nt, *Nicotiana tabacum*; Os, *Oryza sativa*; Pp, *Physcomitrella patens*; Sl, *Solanum lycopersicum*). Scale bar, 10% sequence divergence.

To determine whether the ALAD–SSUII interaction is specific to rice or represents a conserved mechanism across the plant kingdom, we assessed its conservation in plants. We first performed a BlastP search of ALAD homolog proteins from different plant species in UniProt, using OsALAD1 and AtALAD1 as queries [15]. Fifteen homolog sequences from green algae, mosses, and flowering plants were retrieved and analyzed. Multiple sequence alignment demonstrated that ALAD orthologs from different species are highly conserved in their mature protein sequences, with the predicted chloroplast transit peptide regions being the major source of divergence, consistent with the findings of other chloroplast-targeted proteins (Figure S1). The sequence similarity between OsALAD1 and AtALAD1 was 92%, and that between OsALAD1 and the green alga *Chlamydomonas reinhardtii* CrALAD1 was still as high as 76.9%. Phylogenetic analysis demonstrated that all vascular plant ALAD homolog proteins cluster together tightly, consistent with a common evolutionary origin and conserved function (Figure 3).

**Figure 4:**
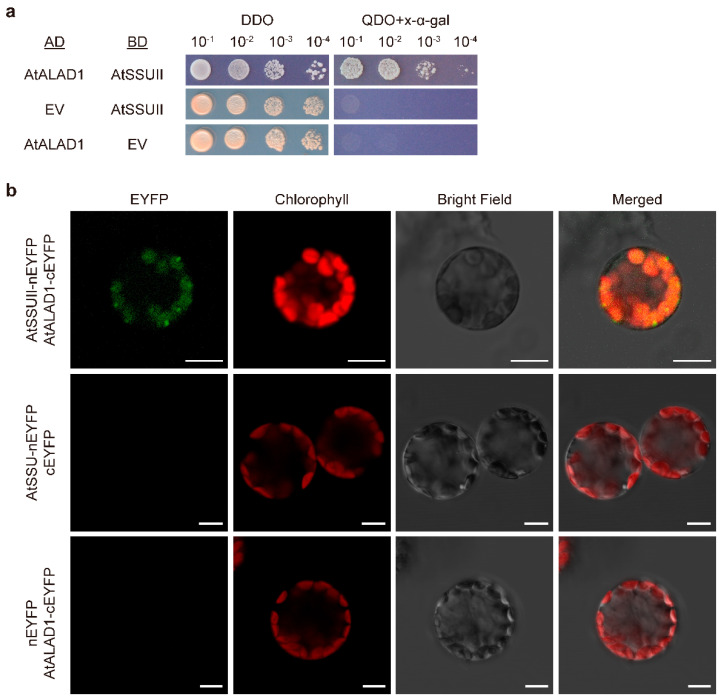
ALAD1 also interacts with SSUII in *Arabidopsis thaliana*. (**a**) Yeast two-hybrid detection. Yeast cells harboring both constructs were spotted on non-selective (DDO) and selective (QDO) medium supplied with X-α-Gal. AD, activation domain of pGAD-T7; BD, DNA-binding domain of pGBK-T7; EV, empty vector. (**b**) BiFC detection in Arabidopsis leaf protoplasts. Co-expressed fusion constructs are indicated. Reconstituted EYFP fluorescence co-localizes with chlorophyll autofluorescence. Scale bars, 10 μm.

We then performed Y2H and BiFC assays to test the interaction between AtSSUII and AtALAD1. Using the same experimental framework as the rice protein assays, yeast cells co-expressing AtALAD1-AD and AtSSUII-BD grew on QDO + X-α-Gal medium and produced blue colonies, demonstrating a positive interaction between the two Arabidopsis proteins (Figure 4a). BiFC assays co-expressing AtSSUII-nEYFP and AtALAD1-cEYFP in Arabidopsis protoplasts produced reconstituted EYFP fluorescence that co-localized with chlorophyll autofluorescence, confirming chloroplast-localized interaction in vivo (Figure 4b). All negative controls produced no detectable EYFP signal (Figure 4b). Taken together, Y2H and BiFC assays in both rice and Arabidopsis provide consistent evidence that the ALAD–SSUII interaction is a conserved feature of chloroplast biology across plant species.

### 2.4. AtSSUII Does Not Alter AtALAD Enzymatic Activity In Vitro, Indicating a Post-Translational Rather than Catalytic Mechanism

**Figure 5:**
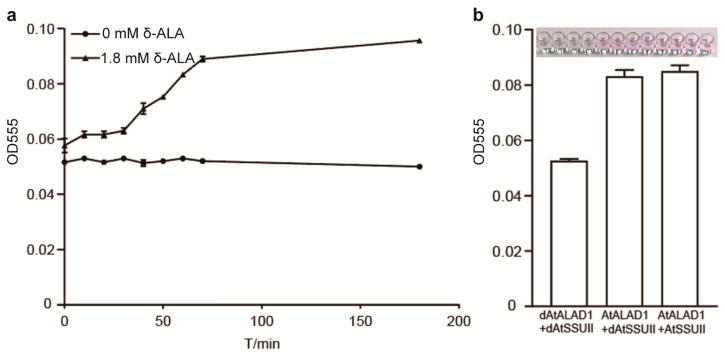
AtSSUII does not alter AtALAD1 enzymatic activity in vitro. (**a**) Time-course analysis of ALAD enzyme activity measured by porphobilinogen accumulation via Ehrlich’s colorimetric assay at OD_555_ nm. Two experimental conditions were monitored: 0 mM ALA (blank control), and 1.8 mM ALA substrate. Error bars represent SD (*n* = 3). (**b**) AtALAD activity was quantified by measuring porphobilinogen formation (OD_555_) under three conditions: both proteins heat-denatured (dAtALDA1 + dAtSSUII), AtSSUII heat-denatured (AtALDA1 + dAtSSUII), and both proteins undenatured (AtALDA1 + AtSSUII). Error bars represent SD (*n* = 3).

To investigate the functional consequence of the ALAD–SSUII interaction on ALAD catalytic activity, we conducted in vitro enzyme activity assays. ALAD activity was quantified by measuring the formation of porphobilinogen (PBG), which reacts with Ehrlich’s reagent to yield a chromogenic product detectable at 555 nm [16]. A time-course experiment was first conducted to establish optimal assay conditions: under 1.8 mM δ-ALA substrate, PBG accumulation reached near-maximum levels within 50–80 min (Figure 5a). Accordingly, 60 min was selected as the standard reaction time for all subsequent quantitative assays.

AtALAD1 enzymatic activity was then quantified under three parallel conditions (Figure 5b): (1) both AtALAD1 and AtSSUII heat-denatured (dAtALAD1 and dAtSSUII; negative control); (2) AtALAD1 un-denatured and AtSSUII heat-denatured; and (3) both proteins un-denatured. All reactions were performed under identical conditions with 1.8 mM substrate, 0.1 mM total protein, in 1× PBS (pH 7.4) at 37 °C for 60 min, with PBG production measured at OD_555_.

No significant difference in OD_555_ values was observed between conditions (2) and (3) (Figure 5b; *p* > 0.05). This result indicates that AtSSUII does not directly affect AtALAD1 enzymatic activity in vitro, indicating that the ALAD–SSUII interaction does not function through direct catalytic modulation of ALAD activity. This is a meaningful mechanistic result: it establishes that the functional consequence of this interaction must be sought at the level of post-translational regulation, such as the recruitment of ALAD to specific subcellular compartments or the stabilization of ALAD within a larger protein complex dedicated to tetrapyrrole biosynthesis [5,17].

### 2.5. Functional Analysis in Transgenic Arabidopsis Plants

To assess the *in planta* consequences of altered ALAD and SSUII expression levels, and to determine whether these proteins regulate each other at the transcriptional level, we generated transgenic *Arabidopsis thaliana* (Col-0) plants overexpressing either *AtALAD1* or *AtSSUII*. Stable transgenic lines were characterized by qRT-PCR.

**Figure 6:**
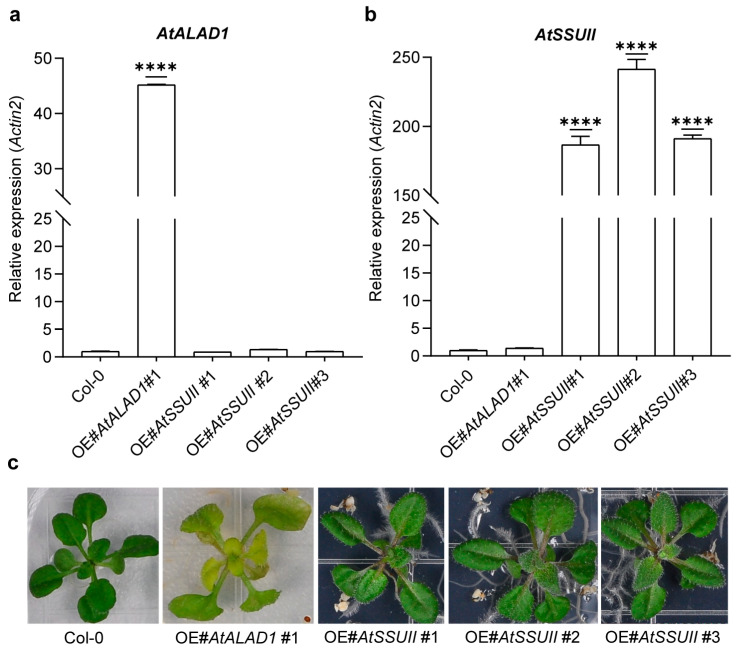
Relative expression levels of *AtALAD1* and *AtSSUII* in transgenic overexpression lines. (**a**) Relative expression levels of *AtALAD1* in *AtALAD1* overexpression line #1 and *AtSSUII* overexpression lines #1–3. (**b**) Relative expression levels of *AtSSUII* in *AtALAD1* overexpression line #1 and *AtSSUII* overexpression lines #1–3. In both panels, *Actin2* was used as a reference. Data represent means ± SD (*n* = 3). Asterisks indicate significant differences between the indicated lines (**** *p* < 0.0001, Student’s *t* test). (**c**) Phenotypic comparison of *AtALAD1* overexpression line #1, *AtSSUII* overexpression lines #1–3, and Col-0 wild-type seedlings.

The *AtALAD1* overexpression line OE#*AtALAD1* #1 showed *AtALAD1* expression levels approximately 45-fold above wild type (Figure 6a). These seedlings exhibited severe chlorosis and subsequently wilted and died after transplanting to soil (Figure 6c). This lethal phenotype is consistent with the known requirement for strict homeostatic control of *ALAD* expression: overaccumulation of ALAD activity leads to uncontrolled flux into the TBP, resulting in accumulation of photoreactive tetrapyrrole intermediates that generate singlet oxygen under illumination and cause severe photooxidative damage [8,9]. Three independent *AtSSUII* overexpression lines (OE#*AtSSUII* #1, #2, and #3) showed *AtSSUII* expression levels of 187-, 242-, and 191-fold above the wild-type level, respectively (Figure 6b). In marked contrast to the *AtALAD1* overexpression line, all three *AtSSUII* overexpression lines displayed no significant phenotypic deviation from WT under the conditions tested (Figure 6c).

Critically, overexpression of *AtALAD1* did not significantly alter *AtSSUII* transcript levels, and overexpression of *AtSSUII* did not significantly alter *AtALAD1* transcript levels either (Figure 6a,b). These results demonstrate that *AtALAD1* and *AtSSUII* do not function as transcriptional regulators of each other, indicating that the ALAD–SSUII interaction operates exclusively at the post-translational level.

## 3. Discussion

The SSUII members, represented by OsSSUII and AtSSUII in this study, were previously found to interact with GGPPSs and to improve their enzymatic activity and specificity in producing GGPP [12]. OsSSUII and the CaSSUII from pepper also function in specifically supplying GGPP for the biosynthesis of chlorophyll and carotenoids, respectively, thereby reducing competition from other metabolic branches for GGPP [12,18]. It is worth noting that, analogous to the branching of GGPP to multiple terpenoid products, PBG generated by ALAD also stands at a metabolic branch point. It feeds not only into chlorophyll biosynthesis but also into the biosynthesis of heme, siroheme, and phytochromobilin [5,19].

The central finding of this study is that we demonstrated the physical interaction between SSUII and ALAD. The interaction was discovered by unbiased Co-IP/LC-MS proteomics and confirmed by different approaches in both rice and Arabidopsis. To our knowledge, this is the first direct protein–protein interaction described between the two upstream biosynthetic branches that jointly supply chlorophyll. We argue that the identification of this interaction is itself a significant advance, independent of the downstream mechanistic details, because it reveals a previously unknown molecular connection at the protein level between the MEP and TBP, two branches whose coordination has long been recognized as biologically important but mechanistically opaque.

What makes this interaction conceptually interesting is its position in the pathway. The TBP and MEP pathways converge at CHLG, which esterifies the porphyrin ring onto phytyl diphosphate. Protein-level connections between the two branches were previously known only near this convergence point: SSUII recruits GGPPS to a thylakoid complex containing CHLG and POR [12], and LIL3 anchors GGR alongside CHLG [17]. The ALAD–SSUII interaction adds a connection at a much earlier stage, where ALAD acts at the second step of the TBP, and SSUII influences GGPP production upstream of GGR. This suggests that cross-pathway communication is not confined to the vicinity of CHLG but begins considerably further upstream, where the two branches first build their respective intermediates. The existence of such an upstream link has important implications: it opens the possibility that the two pathways sense each other’s flux status well before their intermediates physically converge, potentially enabling early-stage coordination of metabolite supply for chlorophyll biosynthesis.

How SSUII might influence ALAD in the chloroplast is not fully resolved by the present data. The finding that the in vitro enzyme assay itself provides mechanistic information: SSUII does not directly modulate ALAD catalytic rate in a purified two-protein system, suggests that the interaction is not mediated through direct allosteric modulation of the ALAD active site. This is itself a meaningful mechanistic result: it rules out a simple kinetic effect and positively points toward post-translational regulation through protein scaffolding or co-localization. Specifically, SSUII may recruit ALAD into the thylakoid-associated multiprotein complex, or position ALAD in proximity to other TBP enzymes, in a manner analogous to how SSUII recruits GGPPS to the same membrane complex. This is consistent with the broader regulatory logic of the chlorophyll biosynthesis network, in which several key regulatory protein–protein interactions, including those involving FLU protein with GluTR and LIL3 with GGR, operate post-translationally to modulate enzyme activity or localization without altering gene transcription [13,17].

The transgenic plant data add a layer of complexity. The lethal chlorosis of OE#AtALAD1 seedlings is consistent with the stringent homeostatic control of TBP flux that has been independently documented [8,9]. This makes it difficult to use gain-of-function genetics to probe the ALAD–SSUII interaction without confounding phenotypes. The absence of any phenotype in *AtSSUII* overexpression lines, combined with the lack of transcriptional cross-regulation in either direction, clarify that there is no a transcriptional relay between the two pathways, and excess SSUII on its own is not sufficient to perturb TBP flux detectably. Whether loss of SSUII reduces ALAD stability, alters its sub-chloroplastic localization, or affects TBP flux under conditions of metabolic demand (high light, de-etiolation, or stress) are questions that remain open. However, the loss of SSUII might also suppress the recruitment of GGPPS to thylakoid membrane for chlorophyll biosynthesis [12].

The cross-species conservation of the interaction suggests it is not a recent or lineage-specific innovation. The high sequence identity of ALAD and SSUII proteins across these species is consistent with the interaction interface having been under purifying selection, though the precise binding surfaces remain to be mapped (Figure S1) [12]. It would be interesting to determine whether the interaction is also present in bryophytes or green algae, which would push its origin back toward the common ancestor of land plants.

Taken together, our data support a model in which SSUII functions as a physical integrator of the two upstream chlorophyll biosynthetic branches, contacting both GGPPS (terpenoid arm) and ALAD (TBP arm) within the chloroplast. How these two interactions are coordinated, i.e., whether SSUII can simultaneously bind both partners, or whether binding is mutually exclusive, is an important unresolved question. Addressing it will require structural studies and in vivo experiments using SSUII mutants that are selectively deficient in one interaction. In the meantime, the present results expand the known protein interaction network linking terpenoid and tetrapyrrole metabolism and identify a new entry point for understanding and potentially manipulating the coordinated regulation of chlorophyll biosynthesis in plants.

## 4. Materials and Methods

### 4.1. Plant Materials and Growth Conditions

Seeds of the wild-type rice cultivar Nipponbare (*Oryza sativa* L. cv. *Nipponbare*) and *Arabidopsis thaliana* (Columbia-0) were used in this study. Rice and Arabidopsis seedlings were grown at 28 °C and 22 °C, respectively, under a light intensity of 120 μmol photons m^−2^ s^−1^ with a 16 h/8 h (light/dark) photoperiod.

### 4.2. Gene Cloning

For both rice and Arabidopsis, RNA was isolated from leaves using the RNAiso reagent (TaKaRa, Shiga, Japan). One microgram of total RNA was used for synthesizing the first-strand cDNA using the HiScript III RT SuperMix for qPCR (+gDNA wiper) (Vazyme, Nanjing, China) following the manufacturer’s instructions. The full-length open reading frame (ORF) of each gene was amplified from the cDNA pool using gene-specific primers. All primers used in this study are listed in Table S2.

### 4.3. Co-Immunoprecipitation and Mass Spectrometry Analysis

Co-immunoprecipitation assay was performed as described with modifications [20]. In brief, 4-week-old rice leaves were homogenized in liquid nitrogen and suspended in dissolving buffer (50 mM HEPES-KOH, pH 7.5, 150 mM NaCl, 2 mM EDTA, 1% Triton X-100, and 1 mM PMSF) at 4 °C for 30 min with gentle mixing. After a 30 min centrifugation at 15,000× *g* at 4 °C, the supernatant was used for the analysis. We first incubated 20 μL of protein A/G agarose (Beyotime, Shanghai, China) with 50 μL anti-OsSSUII antiserum at 4 °C overnight, with a 50-μL pre-immune serum as a negative control [8]. After incubation, the beads were washed 3 times with the dissolving buffer, and then incubated with the protein extract for another 4 h, followed by 5 times of washing using the dissolving buffer. Co-precipitated proteins were separated by SDS-PAGE. After electrophoresis, gels were stained by Coomassie Brilliant Blue R-250 (Sigma-Aldrich, St Louis, MO, USA). Protein bands were excised for liquid chromatography–mass spectrometry analysis using a Nexera UHPLC LC-30A system (Shimadzu, Kyoto, Japan) and a Triple TOF 4600 mass spectrometer (AB Sciex, Framingham, MA, USA) as described [21].

### 4.4. Protein–Protein Interaction Assays

Pairwise Y2H assay was performed as described [22]. The chloroplast transit peptide (cTP) of each protein was predicted using TargetP [23]. ORF encoding the truncated version (without cTP) of each protein was amplified using its full-length cDNA as a template, and then fused with the activation domain (AD) of pGAD-T7 or the DNA-binding domain (BD) of pGBK-T7 (TaKaRa). Previously reported OsGGPPS and OsSSUII were cloned and expressed as a positive control [12]. Empty vectors were used as negative controls. The yeast (*Saccharomyces cerevisiae*) strain AH109 cells transformed with different combinations of plasmids were cultivated and spotted onto non-selective double drop-out (DDO, SD/–Leu/–Trp) plates and selective quadruple drop-out (QDO, SD/–Leu/–Trp/–His/–Ade) plates supplemented with 5-bromo-4-chloro-3-indolyl-β-D-galactopyranoside (X-α-Gal). Images were taken after 3–6 d of cultivation.

For the BiFC assay, protoplasts were prepared from *Arabidopsis thaliana* leaves as described [24]. Full-length ORFs encoding OsSSUII, OsALAD1, AtSSUII, and AtALAD1 were cloned into pSAT1A-cEYFP-N1 or pSAT4A-nEYFP-N1 (ABRC, Ohio State University, Columbus, OH, USA), respectively. A co-expression of OsGGPPS and OsSSUII fusion proteins was used as the positive control. Empty vectors were used as negative controls. Different pairs of plasmids were transformed into the prepared protoplasts as described [12]. Fluorescence signals were observed using a ZEISS 880 laser scanning confocal microscope (Carl Zeiss, Oberkochen, Germany). The EYFP fluorescence was excited with a 488 nm laser, and a detection window at the 500–530 nm range. Chlorophyll autofluorescence signal was excited at 543 nm and detected at the 680–720 nm range.

### 4.5. Multi-Sequence Alignment and Phylogenetic Analysis

Protein sequences of ALAD ortholog sequences from different species were obtained from Uniprot (https://www.uniprot.org/) using the BlastP algorithm. Their accession numbers are listed in Table S1. Sequences were aligned by MUSCLE and manually edited by using GeneDoc (Ver. 2.7, https://genedoc.software.informer.com/2.7/, accessed on 12 February 2026). A Maximum-Likelihood phylogenetic tree was constructed by IQ-TREE with 1000 bootstrap replicates using the JTT+G4 model [25,26].

### 4.6. In Vitro ALAD Enzymatic Activity Assay

ALAD enzymatic activity was quantified by measuring the formation of porphobilinogen (PBG), which reacts with Ehrlich’s reagent to produce a chromogenic product detectable at 555 nm [16]. ORFs encoding the truncated version (without cTP) of AtSSUII and AtALAD1 were amplified and individually cloned into pMAL-C5X for heterologous expression with the maltose-binding protein (MBP) fusion tag. Each of the pMAL-C5X constructs was transformed into *E. coli* Rosetta2(DE3)pLysS cells individually. The expression of target proteins was induced by adding isopropyl β-D-1-thiogalactopyranoside (IPTG) to a final concentration of 0.4 mM at 28 °C for 4 h. MBP-tagged fusion proteins were purified using amylose resin (NEB) according to the manufacturer’s instructions. Purified proteins were confirmed by SDS-PAGE and stained with Coomassie brilliant blue (CBB) R-250 (Sigma-Aldrich, St. Louis, MO, USA).

Enzyme activity was assayed in 200 μL reactions containing 1.8 mM δ-ALA (final concentration) and 0.1 mM total protein in 1× PBS (137 mM NaCl, 2.7 mM KCl, 10 mM Na_2_HPO_4_, 1.8 mM KH_2_PO_4_, pH 7.4) at 37 °C. Reactions were terminated at the indicated times by adding 200 μL of 50% trichloroacetic acid/10% HgCl_2_ (1:1, *v*/*v*). PBG was quantified by addition of 200 μL Ehrlich’s reagent (Macklin, Shanghai, China) followed by incubation for 15 min and absorbance measurement at 555 nm.

### 4.7. Generation of Transgenic Arabidopsis Overexpression Lines

ORFs of AtALAD1 and AtSSUII, each fused to a C-terminal HA tag, were cloned into the pCAMBIA1300-P35S-HSP binary vector under the CaMV *35S* promoter. Binary vectors were transformed into *Agrobacterium tumefaciens* GV3101. Transgenic Arabidopsis plants were generated by the floral dip method [27]. Seeds were selected on Murashige-Skoog (MS) medium containing hygromycin B (40 mg L^−1^). Positive transgenic lines were confirmed by qRT-PCR. Transgenic plants showing significantly elevated transcript levels of the introduced gene were selected from the T_1_ generation. Homozygous T_3_ lines were subsequently obtained through successive self-fertilization and hygromycin resistance screening, and were used for phenotypic characterization and quantitative expression analysis.

### 4.8. RNA Extraction, Reverse Transcription and Gene Expression Analysis

Total RNA was extracted using the RNAiso reagent (TaKaRa, Shiga, Japan), and reverse-transcribed into cDNA with a PrimeScript Double Strand cDNA Synthesis Kit (TaKaRa), following the manufacturer’s instructions. Gene expression levels were quantified by quantitative real-time PCR (qRT-PCR) using SYBR Premix Ex Taq II (TaKaRa) with a Thermal Cycler Dice Real-Time System TP800 (TaKaRa). Transcript abundances were calculated according to the comparative *C*_T_ method [28]. For each sample, at least three biological replicates were analyzed, and each with three repeats. *Actin2* was used as a reference for normalizing gene expression. All primers used in this study are listed in Table S2.

## Figures and Tables

**Table 1:** Proteins co-precipitated with OsSSUII in the Co-IP and LC-MS analysis ^1^.

Name	%Cov	%Cov(50)	%Cov(95)	UniProt ID
Pyridoxal 5′-phosphate synthase subunit	77.96	77.64	75.08	Q8W3D0
Nicotinate-nucleotide pyrophosphorylase	58.49	58.49	56.6	Q0IZS0
δ-Aminolevulinic acid dehydratase	57.04	55.87	55.87	Q5Z8V9
Glyceraldehyde-3-phosphate dehydrogenase	45.77	41.29	41.29	Q7X8A1
GGPPS recruiting protein	36.26	34.21	34.21	Q6ET88
SRP54 domain-containing protein	30.3	25.34	25.34	Q5JK67
CP43	21.35	19.03	19.03	E9KIM9
Rhodanese domain-containing protein	26.1	24.03	24.03	Q6YWR8
LFNR2	51.09	38.8	34.97	Q6ZFJ3
ATP synthase subunit γ	22.35	16.48	13.13	Q84NW1
GST N-terminal domain-containing protein	20.23	19.94	19.94	Q6YZJ0
RubisCO large chain	8.077	8.077	8.077	Q0IPF7
Unknown	10.58	8.466	8.466	Q60E58
Geranylgeranyl diphosphate synthase	16.12	16.12	16.12	Q7XI92
PG binding 1 domain-containing protein	8.719	8.719	8.719	Q7XSJ9
ACT domain-containing protein DS12	11.07	11.07	8.214	Q0J709
Chaperone protein dnaJ A7A	12.08	5.369	5.369	DJA7A
Actin-1	14.06	11.67	11.67	V5K461

^1^ %Cov, sequence coverage; %Cov(50) and %Cov(95), sequence coverage at 50% and 95% confidence thresholds, respectively.

## Data Availability

The original contributions presented in this study are included in the article and Supplementary Materials. Further inquiries can be directed to the corresponding authors.

## Supplementary Materials

The following supporting information can be downloaded at: https://www.mdpi.com/article/10.3390/ijms27104225/s1.
