# Supplementary material for: A Direct ALAD–SSUII Interaction Implies a Potential Link Between Tetrapyrrole and Terpenoid Pathways Toward Chlorophyll Biosynthesis in Plants
Source: Int J Mol Sci. 2026 May 9;27(10):4225. doi: 10.3390/ijms27104225 (PMC13207904; doi:10.3390/ijms27104225)
Supplement: Supplementary file 1 [file ijms-27-04225-s001.zip › ijms-4301870-supplementary.pdf]

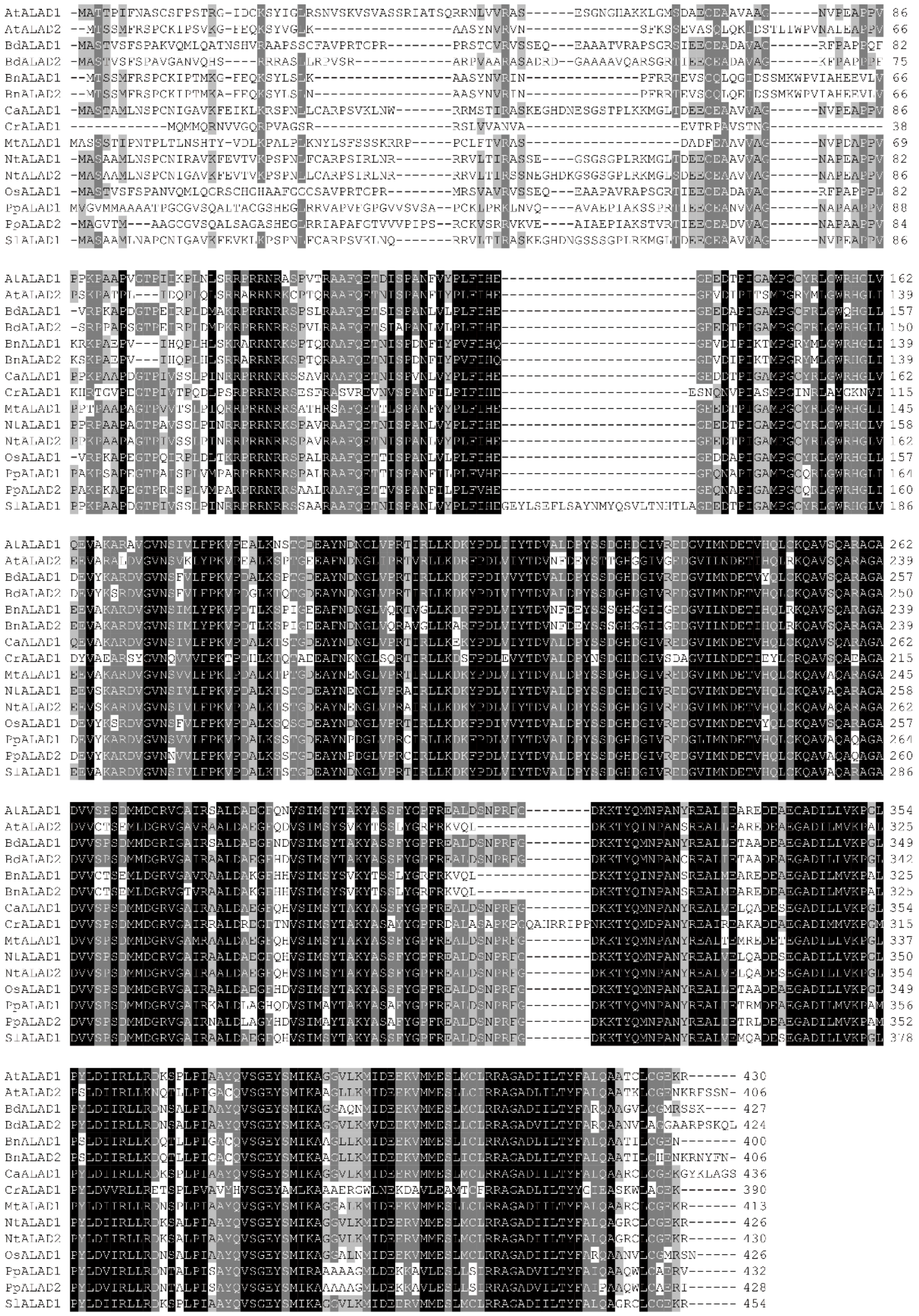

Figure S1. Sequence alignment of plant ALADs.

Sequences were aligned using MUSCLE and manually edited by using GeneDoc.

Table S1. Accession numbers of ALAD ortholog proteins used in sequence comparison and phylogenetic analysis.

Organism	UniProt ID
Arabidopsis thaliana	Q9SFH9 (AtALAD1)
Arabidopsis thaliana	Q94LA4 (AtALAD2)
Brachypodium distachyon	I1GKA8 (BdALAD1)
Brachypodium distachyon	I1HX14 (BdALAD1)
Brassica napus	A0A078FHA3 (BnALAD1)
Brassica napus	A0A078FRN7 (BnALAD2)
Capsicum annuum	A0A1U8FBG2 (CaALAD1)
Chlamydomonas reinhardtii	A8I980 (CrALAD1)
Medicago truncatula	G7J506 (MtALAD1)
Nicotiana tabacum	A0A1S4A3B3 (NtALAD1)
Nicotiana tabacum	A0A1S4AKW2 (NtALAD2)
Oryza sativa	Q5Z8V9 (OsALAD1)
Physcomitrella patens	A0A2K1KNC6 (PpALAD1)
Physcomitrella patens	A9U2X5 (PpALAD2)
Solanum lycopersicum	A0A3Q7ILF6 (SlALAD1)

Table S2. Primers used in this study.

Name	Sequence (5'-3')*
For gene cloning	
OsALAD-F	ATGGCGTCCACCGTCTCCTTCTCCC
OsALAD-R	CTAGTTGGATCGCATGCCGCACAGC
OsSSUII-F	ATGGCTCTCTCCTCCTTCTCCATGT
OsSSUII-R	CTACGGCGTTGTAGCTGCATCCTGC
AtALAD-1F	ATGGCTACTACACCCATCTTTAATG
AtALAD-1R	CTACCGCTTCTCGCCGCACAAACAA
AtSSUII-F	ATGTTGTTTAGTGGTTCAGCGATCC
AtSSUII-R	TCAGAGGGGAAGAAGAAAATGTCGA
For Y2H assay	
AD-OsALAD-F	atatggccatggaggccagtGAGCAGGAGGCGGCGCCGGCGG
AD-OsALAD-R	tgcccacccgggtggaattcCTAGTTGGATCGCATGCCGCAC
AD-AtALAD1-F	atatggccatggaggccagtGCTAGTGAATCCGGCAATGGAC
AD-AtALAD1-R	tgcccacccgggtggaattcCTAGTTGGATCGCATGCCGCAC
AD-OsCHLG-F	atatggccatggaggccagtATGGCCACCTCCCACCTCCTCG
AD-OsCHLG-R	tgcccacccgggtggaattcCTAGTGGCTGGTTGCAAGGGCG
BD-OsSSUII-F	tgcatatggccatggaggccGCCTCCTCTGCCGCCGCCGCCG
BD-OsSSUII-R	cgacggatccccgggaattcCTACGGCGTTGTAGCTGCATCC
BD-AtSSUII-F	tgcatatggccatggaggccTCTTCATCCTCATCTGCCCCGG
BD-AtSSUII-R	cgacggatccccgggaattcTCAGAGGGGAAGAAGAAAATGT
For BiFC assay	
pSAT4A-OsSSUII-nYFP-F	agatctcgagctcaagcttcATGGCTCTCTCCTCCTTCTCCA
pSAT4A-OsSSUII-nYFP-R	ccgcggtaccgtcgactgcaCGGCGTTGTAGCTGCATCCTGC
pSAT1A-OsALAD-cYFP-F	agatctcgagctcaagcttcATGGCGTCCACCGTCTCCTTCT
pSAT1A-OsALAD-cYFP-R	ccgcggtaccgtcgactgcaGTTGGATCGCATGCCGCACAGC
pSAT1A-OsGGPPS-cYFP-F	agatctcgagctcaagcttcATGGCTGCCTTCCCCCCGCTCG
pSAT1A-OsGGPPS-cYFP-R	ccgcggtaccgtcgactgcaGTTCTGCCGATAGGCAATATAA
pSAT4A-AtSSU-nYFP-F	agatctcgagctcaagcttcATGTTGTTTAGTGGTTCAGCGA
pSAT4A-AtSSU-nYFP-R	ccgcggtaccgtcgactgcaGAGGGGAAGAAGAAAATGTCGA
pSAT1A-AtALAD1-cYFP-F	agatctcgagctcaagcttcATGGCTACTACACCCATCTTTA
pSAT1A-AtALAD1-cYFP-R	ccgcggtaccgtcgactgcaCCGCTTCTCGCCGCACAAACAA
For Overexpression	
pCAMBIA1300-p35S-AtALAD1-HSP-F	tttcaccatttacgaacgatagATGGCTACTACACCCATCTT
pCAMBIA1300-p35S-AtALAD1-HSP-R	tcttcatcttcatatCTAAGCGTAATCTGGTACGTCGTATG GGTACCGCTTCTCGCCGC
pCAMBIA1300-p35S-AtSSUII-HSP-F	tcaccatttacgaacgatagATGTTGTTTAGTGGTTCAGC
pCAMBIA1300-p35S-AtSSUII-HSP-F	tcttcatcttcatatCTAAGCGTAATCTGGTACGTCGTATG GGTAGAGGGGAAGAAGAA
For qRT-PCR	
AtALAD1-qRT-F	ACAAGCTGTTTCCCAGGCTC
AtALAD1-qRT-R	GCTTCACGGAAAGGACCGTA

AtSSUII-qRT-F	AGCCACCATCCTCAGACTCA
AtSSUII-qRT-R	CATTCACCCATGGCTCCGAA
Actin2-qRT-F	CCAACATATGCATCCTTCTGGTTCATCCCA
Actin2-qRT-R	TGGCTGAGGCTGATGATATTCAACCAATCG

* Fragments with nucleotides in lower case letters are designed for cloning through homologous recombination.
